# Resveratrol Affects Insulin Signaling in Type 2 Diabetic Goto-Kakizaki Rats

**DOI:** 10.3390/ijms22052469

**Published:** 2021-02-28

**Authors:** Katarzyna Szkudelska, Marzanna Deniziak, Maciej Sassek, Ignacy Szkudelski, Wojciech Noskowiak, Tomasz Szkudelski

**Affiliations:** 1 Department of Animal Physiology, Biochemistry and Biostructure, Poznan University of Life Sciences, Wolynska 35, 60-637 Poznan, Poland; katarzyna.szkudelska@up.poznan.pl (K.S.); maciej.sassek@up.poznan.pl (M.S.); 2 College of Natural Sciences, University of Rzeszów, Pigonia 1, 35-310 Rzeszów, Poland; deniziak@univ.rzeszow.pl; 3 Independent Software Developer, 60-637 Poznan, Poland; kaignacy@gmail.com (I.S.); wojtek.noskowiak@gmail.com (W.N.)

**Keywords:** resveratrol, type 2 diabetes, insulin, insulin receptor

## Abstract

Resveratrol is a biologically active diphenolic compound exerting multiple beneficial effects in the organism, including anti-diabetic properties. This action is, however, not fully elucidated. In the present study, we examined effects of resveratrol on some parameters related to insulin signaling, and also on diabetes-associated dysregulation in Goto-Kakizaki (GK) rats with congenital type 2 diabetes. Resveratrol was given at the dose of 20 mg/kg b.w. for 10 weeks. It was shown that the expression and phosphorylation levels of insulin receptor in the skeletal muscle of GK rats were significantly decreased, compared with control animals. However, these changes were totally prevented by resveratrol. Liver expression of the insulin receptor was also reduced, but in this case, resveratrol was ineffective. Resveratrol was also demonstrated to significantly influence parameters of insulin binding (dissociation constant and binding capacity) in the skeletal muscle and liver. Moreover, it was shown that the expression levels of proteins related to intracellular glucose transport (GLUT4 and TUG) in adipose tissue of GK rats were significantly decreased. However, treatment with resveratrol completely abolished these changes. Resveratrol was found to induce normalization of TUG expression in the skeletal muscle. Blood levels of insulin and GIP were elevated, whereas proinsulin and GLP-1 diminished in GK rats. However, concentrations of these hormones were not affected by resveratrol. These results indicate that resveratrol partially ameliorates diabetes-associated dysregulation in GK rats. The most relevant finding covers the normalization of the insulin receptor expression in the skeletal muscle and also GLUT4 and TUG in adipose tissue.

## 1. Introduction

Resveratrol (3,5,3′-trihydroxystilbene) is a naturally occurring bioactive compound present in different plant species. It is also found in considerable amounts in red wine [1]. Resveratrol ingestion by animals and humans is associated with numerous favorable effects. These effects were shown under various pathological conditions, including diabetes. Results of animal studies and also human clinical trials indicate that resveratrol has a great potential to alleviate dysregulation occurring in type 2 diabetes [2,3]. The pivotal symptoms of type 2 diabetes are insulin resistance, hyperglycemia, and a progressive failure of pancreatic β-cells. However, this is, in fact, a much more complex disease, and diabetes-associated changes cover various tissues. The insulin-sensitive tissues, such as the skeletal muscle, liver, and adipose tissue, are primarily affected. It is also known that chronically elevated blood glucose levels largely contribute to the dysfunction of other tissues and organs and also to the development of diabetic complications [4]. It is recently well established that hormonal and metabolic changes occurring in type 2 diabetes are strongly associated with inflammatory and oxidative stress, which additionally worsens the diabetic state [5]. Resveratrol is known to exert pleiotropic action, and its anti-diabetic potential is complex. Results of numerous studies have provided evidence that in animals with experimentally induced type 2 diabetes, resveratrol treatment is associated with the decline in blood glucose levels. Better glycemic control is largely linked with the reduction of insulin resistance and the resulting increased glucose uptake by the skeletal muscle. Resveratrol was also shown to improve the structure and the endocrine function of pancreatic β-cells [2,6]. The anti-diabetic effects of resveratrol are partly attributed to its anti-oxidative [7] and anti-inflammatory [8] properties. Resveratrol is explored in the context of its anti-diabetic action also in humans. This is an important issue, given that diabetes affects more than 5% of people worldwide with increasing morbidity. Moreover, diabetes is associated with numerous complications [4]. Results of human studies and clinical trials indicate that resveratrol therapy markedly reduces blood glucose levels and improves insulin action in patients with type 2 diabetes [9,10,11,12,13,14]. Moreover, it was shown that resveratrol alleviates the inflammatory and oxidative stress in people with type 2 diabetes [2,10]. Resveratrol may also reduce diabetes complications, such as diabetic ulcer size [12]. However, the beneficial effects of resveratrol related to blood glucose levels and insulin resistance were not confirmed in other studies on humans with type 2 diabetes [2,15,16]. Thus, results concerning resveratrol action in humans with type 2 diabetes are very differentiated; some research shows that resveratrol is effective, however, others indicate a lack of effect. This may be partially related to different doses of resveratrol used in the studies. The available data indicate that resveratrol has a potential to alleviate symptoms of diabetes in humans, however, further clinical trials are required to elucidate the discrepancies [2,17]. In spite of much research, the therapeutic potential of resveratrol in diabetes is still not fully explored, and some new aspects may be discovered. Our recent results provided evidence that resveratrol markedly alleviates many diabetes-associated symptoms in GK rats, a model of type 2 diabetes [18,19,20]. GK rats are non-obese, however, they display mild hyperglycemia, substantial insulin resistance, hyperinsulinemia, and a progressive failure of pancreatic β-cells [21]. It was shown that resveratrol treatment of GK rats improves, among other, glucose tolerance. This positive effect is largely attributed to its influence on the skeletal muscle. In the skeletal muscle of GK rats, lipid accumulation was demonstrated to be substantially augmented. This is associated with reduced intramuscular glucose transport. Moreover, increased lipid accumulation was shown to be accompanied by the excessive expression and phosphorylation of acetyl-CoA carboxylase (ACC) and protein kinase B (Akt). However, resveratrol therapy prevented lipid accumulation and markedly reduced muscle ACC and Akt expression [18]. Metabolic changes in GK rats are accompanied by many symptoms of inflammation and oxidative stress. However, these disturbances were also alleviated by resveratrol [19]. These results clearly indicate that resveratrol effectively reduces plenty of pivotal diabetes-associated disorders in GK rats. However, some aspects addressing its action in this animal model are not elucidated. The present study aimed to better elucidate the anti-diabetic properties of resveratrol in GK rats. We mainly focused on parameters related to insulin signaling, levels of some hormones, and expression of proteins involved in the intracellular glucose transport.

## 2. Results

### 2.1. Effects of Resveratrol on FIGR, HOMA-IR, QUICKI, and Hormone Levels

**Figure 1:**
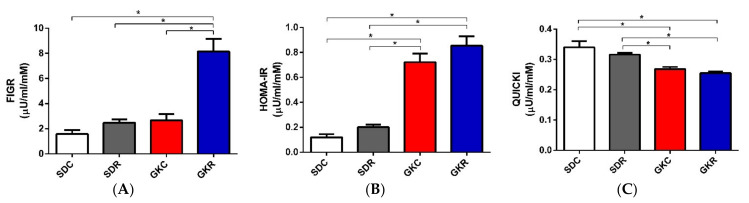
Effects of resveratrol on fasting insulin/glucose (FIGR) (**A**), homeostatic model assessment—insulin resistance (HOMA-IR) (**B**), and quantitative insulin sensitivity check index (QUICKI) (**C**) in SD and GK rats. To calculate these indices, rats were fasted 12 h before taking blood for measurement glucose and insulin levels. SDC—Sprague-Dawley control rats, SDR—Sprague-Dawley rats treated with resveratrol, GKC—Goto-Kakizaki control rats, GKR—Goto-Kakizaki rats treated with resveratrol. Results represent the means ± SEM from 10 rats. *—Differences statistically significant (*p* < 0.05).

In the present study, many diabetes-associated parameters were compared in GK and SD rats to determine the potential action of resveratrol. Indices related to insulin resistance, such as fasting insulin/glucose ratio (FIGR), homeostatic model assessment—insulin resistance (HOMA-IR), and quantitative insulin sensitivity check index (QUICKI), were measured in fasted animals. Results of our study have shown that values of HOMA-IR were significantly increased (*p* < 0.05), QUICKI decreased (*p* < 0.05), and FIGR remained unchanged in diabetic rats, compared with the control. Resveratrol ingestion by GK rats was associated with a significant (*p* < 0.05) rise in FIGR, however, HOMA-IR and QUICKI remained unchanged (Figure 1).

Apart from these indices, all other parameters were determined in non-fasted rats. It was shown that concentrations of blood insulin were significantly increased (*p* < 0.05), proinsulin decreased (*p* < 0.05), and glucagon unchanged in GK rats. In the present study, blood levels of these hormones were not affected in rats receiving resveratrol (Table 1).

Along with changes in insulin and proinsulin, GK rats were shown to display a substantial dysregulation in blood incretin levels. Concentrations of GIP were significantly reduced (*p* < 0.05), and GLP-1 slightly increased in these animals. However, resveratrol treatment did not significantly influence neither GIP nor GLP-1 (Table 1).

### 2.2. Effects of Resveratrol on Expression of Insulin Receptor and on Insulin Binding

**Figure 2:**
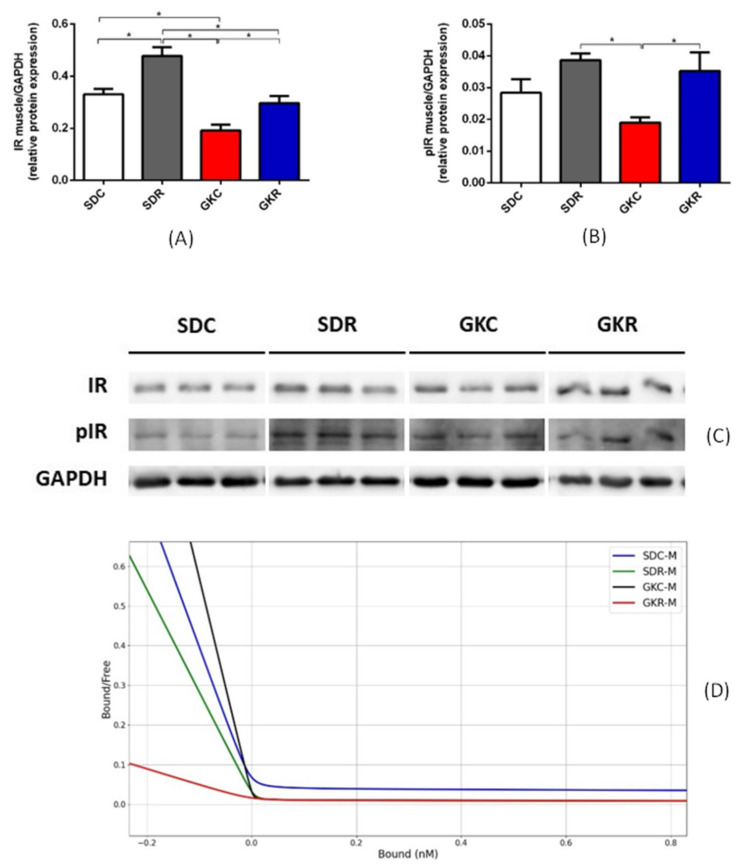
Effects of resveratrol on expression and phosphorylation of insulin receptor and on insulin binding in the skeletal muscle of SD and GK rats. Expression of insulin receptor (**A**), phosphorylation of insulin receptor (**B**), Western blot immunodetection with antibodies to the indicated proteins, GAPDH expression used as a loading control. Protein levels relative to GAPDH quantified by densitometry. Results are representative of at least two independent experiments (**C**), Scatchard plot showing insulin binding to insulin receptor (**D**). Bound—insulin bound to insulin receptor, Free—free insulin. SDC—Sprague-Dawley control rats, SDR—Sprague-Dawley rats treated with resveratrol, GKC—Goto-Kakizaki control rats, GKR—Goto-Kakizaki rats treated with resveratrol. Results represent the means ± SEM from 10 rats. In the case of studies concerning insulin binding, tissues from 2–3 rats were pooled and results were expressed as the means ± SEM from 4 separate determinations *—Differences statistically significant (*p* < 0.05).

Western blot analysis have shown that the expression levels of insulin receptor in the skeletal muscle of GK rats were significantly lower (*p* < 0.05), compared with non-diabetic animals. However, resveratrol therapy was associated with a significant rise in its expression in diabetic rats (*p* < 0.05) to values comparable with the control group (Figure 2, Figure S1). Moreover, the phosphorylation levels of insulin receptor tended to be reduced in GK rats, and resveratrol caused a significant (*p* < 0.05) increase in receptor phosphorylation (Figure 2, Figure S1).

**Figure 3:**
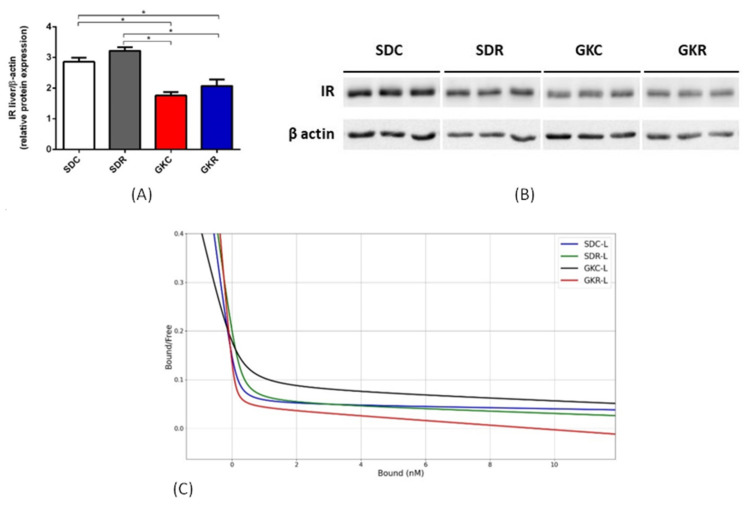
Effects of resveratrol on the expression of insulin receptor and on insulin binding in the liver of SD and GK rats. Expression of insulin receptor (**A**), Western blot immunodetection with antibodies to the indicated proteins, β-actin expression used as a loading control. Protein levels relative to β-actin quantified by densitometry. Results are representative of at least two independent experiments (**B**), Scatchard plot showing insulin binding to the insulin receptor (**C**). Bound—insulin bound to the insulin receptor, Free—free insulin. SDC—Sprague-Dawley control rats, SDR—Sprague-Dawley rats treated with resveratrol, GKC—Goto-Kakizaki control rats, GKR—Goto-Kakizaki rats treated with resveratrol. Results represent the means ± SEM from 10 rats. In the case of studies concerning insulin binding, tissues from 2–3 rats were pooled and results were expressed as the means ± SEM from 4 separate determinations. *—Differences statistically significant (*p* < 0.05).

Similarly to the muscle tissue, liver expression of the insulin receptor in GK rats was also significantly decreased (*p* < 0.05). However, in this case, expression was not markedly changed by resveratrol (Figure 3, Figure S2).

Analysis of insulin binding to its receptor has shown that in the skeletal muscle of GK rats, resveratrol significantly (*p* < 0.05) reduced Kd_1_ and Kd_2_, compared with non-treated diabetic animals. It was also demonstrated that resveratrol significantly (*p* < 0.05) increased R_1_ and R_2_ in the muscle tissue derived from GK rats (Table 2, Figure 2).

In the present study, parameters related to liver insulin binding markedly differed from values shown for the skeletal muscle. Our results have revealed that resveratrol therapy was associated with a significant (*p* < 0.05) increase in Kd_1_ and Kd_2_ in the liver of GK rats. Moreover, in the group of GK rats receiving resveratrol, R_1_ and R_2_ in the liver tissue were significantly decreased (*p* < 0.05) (Table 2, Figure 3).

### 2.3. Effects of Resveratrol on Expression of GLUT4 and TUG

**Figure 4:**
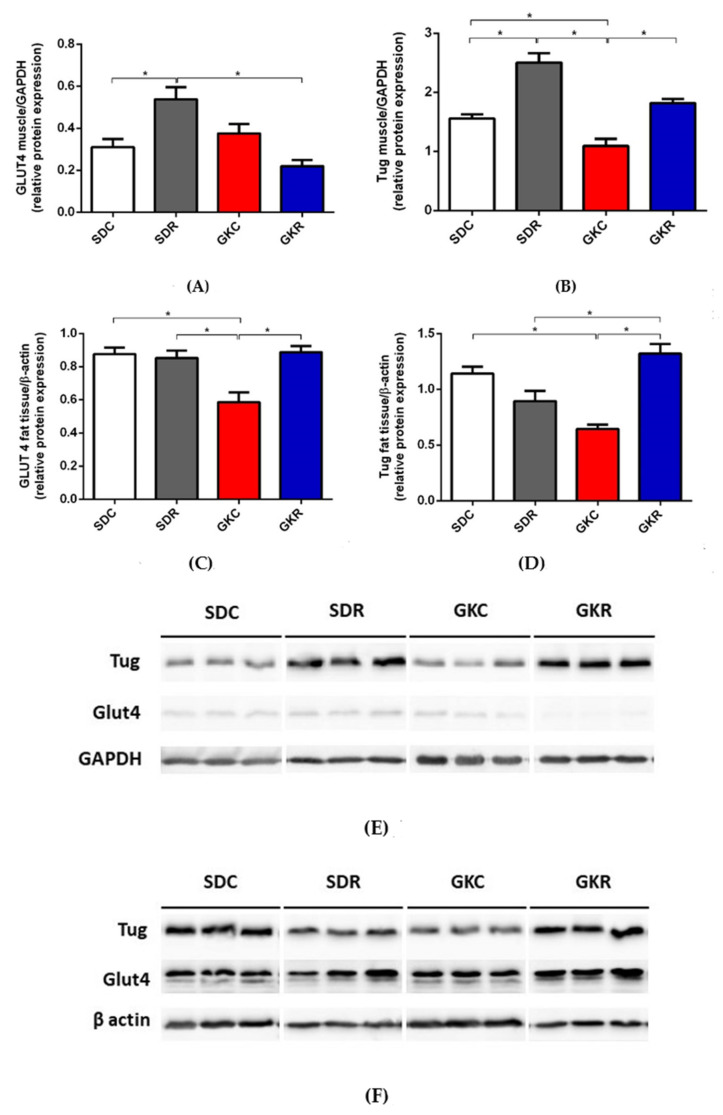
Effects of resveratrol on the expression of GLUT4 and TUG in the skeletal muscle and fat tissue of SD and GK rats. The skeletal muscle GLUT4 (**A**) and TUG (**B**), fat tissue GLUT4 (**C**), and TUG (**D**). Western blot immunodetection with antibodies to the indicated proteins, GAPDH or β-actin expression used as a loading control. Protein levels relative to GAPDH or β-actin quantified by densitometry. Results are representative of at least two independent experiments. The skeletal muscle (**E**) and fat tissue (**F**). SDC—Sprague-Dawley control rats, SDR—Sprague-Dawley rats treated with resveratrol, GKC—Goto-Kakizaki control rats, GKR—Goto-Kakizaki rats treated with resveratrol. Results represent the means ± SEM from 10 rats. *—Differences statistically significant (*p* < 0.05).

In the present study, the expression levels of GLUT4 in the skeletal muscle of control and diabetic rats did not differ significantly. On the other hand, TUG expression in the muscle tissue of GK rats was significantly decreased (*p* < 0.05). However, resveratrol treatment restored its expression to values found in SD rats (Figure 4, Figure S3A).

It was also found that the expression levels of GLUT4 and TUG in adipose tissue of GK rats was significantly lower compared with control animals (*p* < 0.05). However, our results have shown that resveratrol therapy was associated with normalized expression of both proteins in diabetic rats (*p* < 0.05) (Figure 4, Figure S3B).

## 3. Discussion

In GK rats, diabetes-associated dysregulation covers numerous hormones, signaling molecules, enzymes, and other relevant factors. Our recent research has revealed that many of these alterations are substantially alleviated by resveratrol therapy [18,19,20]. It was previously shown that GK rats display increased both fasting blood glucose and insulin levels compared with control animals [18]. Therefore, calculated in the present study, values of HOMA-IR were higher, QUICKI lower, and FIGR unchanged in diabetic rats. Similar differences between GK and control rats were found in other research [22]. Resveratrol treatment of diabetic rats caused a substantial decrease in fasting blood glucose levels with a concomitant additional increase in insulinemia [18]. These changes resulted in a significant rise in FIGR in diabetic rats subjected to resveratrol therapy, however, HOMA-IR and QUICKI remained unchanged. Given that fasting hyperglycemia is a hallmark of insulin resistance, resveratrol-evoked reduction in blood glucose levels indicates improved glucose tolerance in diabetic rats. 

It was shown that resveratrol treatment increased fasting [18] and non-fasting insulin levels in GK rats. The concentration of blood insulin depends on both the rate of hormone secretion and its elimination. The liver is responsible for a large part of circulating insulin elimination. This is associated with the hepatic activity of the insulin-degrading enzyme (IDE). It is possible that resveratrol increases blood insulin in GK rats by inhibiting IDE, which leads to reduced hepatic clearance of this hormone. However, data on the effects of resveratrol on liver IDE, to confirm this assumption are lacking. On the other hand, a large body of evidence indicates that resveratrol improves the structure and function of pancreatic islets in diabetic rats [2,6]. This effect was also demonstrated in GK rats treated with resveratrol [18]. These results indicate that elevated blood insulin levels in GK rats subjected to resveratrol therapy are due to increased secretion of this hormone. GK rats develop a progressive failure of pancreatic β-cells, and blood insulin levels decline with age reaching values below physiological [21,23]. Thus, a rise in insulinemia shown in fasted rats treated with resveratrol [18] confirms its protective effects on β-cells. The beneficial effect of resveratrol on islet structure may be attributed to multiple factors. Under physiological conditions, gut-derived incretin hormones (GIP and GLP-1) play a pivotal role. GIP and GLP-1 are largely implicated in the promotion of glucose-induced insulin secretion and also in the preservation of β-cell functionality. However, diabetes is usually accompanied by abnormal incretin secretion and action [24]. In line with this notion, blood levels of GIP were reduced, whereas GLP-1 elevated in GK rats, compared with the control group. It was, however, shown that concentrations of the incretin hormones were not affected by resveratrol. This suggests that the favorable effect of resveratrol on the structure of pancreatic islets in GK rats [18] is irrespective of GIP and GLP-1. It may be largely associated with resveratrol’s anti-oxidant and anti-inflammatory effects in GK rats [19].

In our present study, blood insulin levels determined in non-fasted diabetic rats were markedly increased, and proinsulin reduced, compared with the control group. However, these hormones were not significantly affected by resveratrol. Moreover, we did not observe any differences in blood glucagon levels in diabetic and control rats, which is in line with results showing that pancreatic α-cells in GK rats are much less affected [21].

Insulin resistance developing in GK rats is strongly associated with abnormal insulin signaling in the metabolically active tissues [18,25]. This especially covers the skeletal muscle, which plays a relevant role in whole-body glucose homeostasis. Many defects have been implicated in skeletal muscle insulin resistance in GK rats. One of them is disturbed phosphorylation of the insulin signaling pathway proteins (Akt and GSK-3β) [25]. Under physiological conditions, these proteins undergo phosphorylation in response to insulin, enabling the transition of the signal. However, in GK rats, their phosphorylation is excessive, which is largely linked with the impaired insulin action. Our previous research has shown that defects covering both protein phosphorylation and metabolic disturbances are markedly alleviated by resveratrol [18]. In the present study, we focused on the insulin receptor, which is the first step of the insulin signaling pathway. It was shown that the expression level of this receptor in the skeletal muscle of GK rats was decreased. However, resveratrol treatment completely abolished this decrease and recovered its expression to control values. Under physiological conditions, insulin receptors, along with its intracellular substrates (IRS), undergo phosphorylation as a result of insulin action. However, in the skeletal muscle of GK rats, phosphorylation of both insulin receptor and IRS-1 was previously found to be reduced, which largely contributes to the impaired insulin signaling [26,27,28]. This reduction results from the exaggerated expression and activity of protein-tyrosine phosphatase in GK rats, an enzyme catalyzing dephosphorylation of insulin receptor and IRS-1 [28,29]. In line with these findings, in our present study, the insulin receptor’s phosphorylation level in the skeletal muscle of GK rats was reduced, while resveratrol caused its normalization. This positive effect may be due to the inhibition by resveratrol of protein-tyrosine phosphatase. Such an action of resveratrol is known to occur in other animal models with insulin resistance [30]. It should be emphasized that the excessive phosphorylation of ACC and Akt in the muscle tissue of GK rats covers serine residue [18], while reduced phosphorylation level of insulin receptor and IRS-1 was shown at tyrosine. This difference in the phosphorylation sites explains why the up-regulation of protein-tyrosine phosphatase in the skeletal muscle of GK rats resulted in reduced phosphorylation of insulin receptor and IRS-1 with a concomitant increased phosphorylation levels of ACC and Akt. The beneficial effects of resveratrol addressing expression and phosphorylation of insulin receptor may be supposed to improve insulin action in GK rats.

Apart from the expression level of the insulin receptor, effects of resveratrol on parameters related to insulin binding, such as dissociation constant and binding capacity, were also assessed. Dissociation constant refers to the receptor affinity and is inversely correlated with the affinity, whereas binding capacity indicates the total number of insulin receptors. Two classes of insulin receptors, which are present in tissues, i.e., high-affinity insulin receptors (HAIRs) and low-affinity insulin receptors (LAIRs), were taken into account. It is also known that various interactions occur between receptors [31]. In our study, resveratrol reduced the dissociation constant and increased binding capacity for both classes of insulin receptors in the skeletal muscle of GK rats. This suggests that resveratrol increased the affinity of insulin receptors and concomitantly increased their number. GK rats were shown to have an unchanged expression of GLUT4 in the skeletal muscle, and resveratrol did not significantly affect its expression. On the other hand, expression of the other important protein indirectly involved in glucose transport into the muscle cells, i.e., TUG, was markedly decreased in GK rats. Under physiological conditions, TUG protein binds to GLUT4 and regulates its trafficking. However, diabetes is usually associated with dysregulation of GLUT4, TUG, and other proteins participating in glucose transport into adipocytes and muscle cells [32,33]. Moreover, TUG is thought to act as a major insulin-regulated step for GLUT4 translocation. In order to stimulate glucose uptake via GLUT4, insulin triggers TUG endoproteolytic cleavage. It is also known that TUG depletion reduces GLUT4 protein stability [34]. It was, however, shown that resveratrol therapy was associated with normalization of TUG expression in the skeletal muscle of GK rats.

Compared with the skeletal muscle, liver of GK rats is known to be less affected. Liver lipid accumulation may be unchanged or only moderately increased [18,25]. Results of the present study have shown that liver expression of insulin receptor in GK rats was decreased. A similar decrease was previously found in hyperinsulinemic GK rats [35]. In our present study, liver expression of the insulin receptor was, however, not altered by resveratrol. Bisbis et al. [36] have reported reduced insulin binding by liver insulin receptors in GK rats. Our results revealed that resveratrol increased dissociation constant and reduced binding capacity in the liver of diabetic rats. This suggests reduced affinity and reduced number of insulin receptors in the liver tissue. The binding capacity of the insulin receptor is affected by many factors. One of the pivotal factors is insulin supply. The excessively increased concentration of this hormone reduces the binding capacity as a result of the negative interaction [31]. In our study, resveratrol caused a significant increase in blood insulin in fasted rats [18] and also a slight increase in non-fasted animals. Moreover, it is known that insulin secreted by the pancreatic β-cells reaches firstly the liver, in which a large part of the hormone is degraded. This causes the effects of insulin in the liver to be greater compared with other organs. Thus, the enhanced insulin action in the liver tissue may be associated with the reduction of insulin binding capacity in GK rats treated with resveratrol. This is inversely to effects elicited by resveratrol in the skeletal muscle. Insulin binding is known to be tissue-specific, and in addition, substantial differences related to binding parameters are often found [31,37].

Although GK rats are non-obese, the adipose tissue of these animals displays many symptoms strongly associated with insulin resistance. One of them is inflammation [38], which is manifested by the up-regulation of some genes related to chronic inflammation [39] and also by an increased content of inflammatory markers [19]. In our present study, in line with the previous findings [29], GK rats were shown to have significantly reduced expression of GLUT4 in adipose tissue. Importantly, this decrease was totally abolished by resveratrol therapy. It was demonstrated that the expression levels of GLUT4 in adipose tissue of GK rats treated with resveratrol and in control animals were similar. The effect elicited by resveratrol in adipose tissue differed compared with changes induced in the skeletal muscle. This is in accord with the results showing that in animal models with insulin resistance, the response to resveratrol treatment may be tissue-specific [19,40]. Along with changes covering GLUT4 in adipose tissue of GK rats, the expression level of TUG was also shown to be markedly reduced. However, in diabetic rats ingesting resveratrol, TUG expression was also restored to the levels observed in the control group. Intracellular glucose transport via GLUT4 is complex, and many proteins and signaling molecules have been implicated. Although GLUT4 is the major glucose transporter in both adipose tissue and the skeletal muscle, many tissue differences related to glucose transport were shown under physiological and pathological conditions. One of them is the greater ability of insulin to redistribute GLUT4 in adipose tissue of healthy animals. Insulin resistance is also associated with disturbances, which are not identical in adipose tissue and the skeletal muscle. It is, among others, known that in insulin resistance, glucose transport into both tissues is reduced, however, adipose tissue is less affected [32,33]. In accord with these data, results of the present study have shown differences in expression of GLUT4 and TUG in adipose tissue and the skeletal muscle. The normalization of GLUT4 and TUG expression in fat tissue of diabetic rats is particularly interesting, given that resveratrol did not alleviate the fat tissue inflammatory state [19]. The link between adipose tissue inflammation and its dysfunction in diabetes is relatively well established [38]. These results strongly suggest that the beneficial action of resveratrol on GLUT4 and TUG expression is irrespective of the adipose tissue inflammatory state.

Our study has some limitations. We could determine only the expression of liver insulin receptors without the level of its phosphorylation. Moreover, in experiments concerning insulin binding, tissues had to be pooled to get measurable results. On the other hand, in spite of the abundance of data addressing the anti-diabetic effects of resveratrol, our research was performed on GK rats, in which the action of resveratrol is poorly explored, and the obtained results are new. This may be considered a strength of our study.

## 4. Materials and Methods

### 4.1. Animals and Treatment

Animals used in the experiment were purchased from Taconic Biosciences Inc. (Germantown, MD, USA). Three-week-old male Goto-Kakizaki (GK) rats and age-matched male Sprague-Dawley (SD) rats were kept in an air-conditioned room at constant temperature 21 ± 1 °C in 12/12 h dark-light cycle and were fed a diet recommended for GK rats (Rodent NIH-31 M Auto diet, Zeigler Bros. Inc., Gardners, PA, USA) containing 18% crude protein, 5% crude fat, 5% crude fiber and gross energy at 4.02 kcal/g and had free access to tap water. After 3 weeks of the adaptation period, animals were randomly divided into 4 groups of 10 animals each: GK rats receiving vehicle (GKC), GK rats receiving resveratrol (GKR), SD rats receiving vehicle (SDC), and SD rats receiving resveratrol (SDR). Rats were placed in cages of 2–3 individuals, and body weight was checked twice a week during the entire experiment. Resveratrol (Cayman, Ann Arbor, MI, USA) or vehicle was administered to rats from the beginning of the 6th week of life. It was dissolved in 0.5% carboxymethylcellulose (Sigma-Aldrich, St. Louis, MO, USA) and administered intragastrically at the dose 20 mg/kg b.w. by gavage once a day for 10 weeks. Doses of resveratrol and time of its administration in animal studies addressing diabetes are very differentiated. Based on the literature data, it can be said that the effective amounts of resveratrol in rats and mice with experimentally induced diabetes are often between 5 and 50 mg per kg body weight. Resveratrol is usually given for a few weeks [41]. It is also known that the toxicity of resveratrol is relatively low. Other studies have shown that oral administration of resveratrol to rats for 28 days at the dose of 300 mg/kg does not induce toxic effects [41,42]. Therefore, we administered 20 mg of resveratrol per kg, supposing that this dose is effective and safe. Moreover, our study has shown that the same amount of resveratrol and time of treatment did not affect activities of enzymes related to organ failure [19]. This means that the dose of resveratrol used in the present study is not harmful.

### 4.2. Determination of FIGR, HOMA-IR, QUICKI, and Tissue Sampling

After 8 weeks of the experiment, rats were fasted overnight (12 h), and blood samples were taken from the tail vein to determine blood glucose and insulin and to calculate fasting insulin/glucose ratio (FIGR), homeostatic model assessment—insulin resistance (HOMA-IR) and quantitative insulin sensitivity check index (QUICKI). HOMA-IR was calculated using the following formula: Fasting glucose x fasting insulin /22.5, in the case of QUICKI, the following formula was used: 1/ log (fasting insulin) + log (fasting glucose). The results concerning blood insulin and glucose levels in fasted rats were published in our previous study [18]. In the present research, these values were used only to calculate FIGR, HOMA-IR, and QUICKI. Concentrations of fasting insulin were the following (μU/mL): 8.75 (SDC), 14.5 (SDR), 26.2 (GKC), and 53.0 (GKR). Blood glucose levels in fasted rats were the following (mM): 5.5 (SDC), 5.6 (SDR), 11.1 (GKC), and 6.5 (GKR) [18]. All other hormones and parameters included in the study were measured in non-fasted animals.

After 10 weeks of the treatment with resveratrol or vehicle, non-fasted animals were killed by decapitation and their tissues (blood, skeletal muscle, liver, visceral fat tissue) were sampled and stored for analysis (−80 °C).

The permission (no. 48/2016; date of approval 1 July 2016) of the Local Ethical Commission for Investigations on Animals in Poznan was obtained to conduct the experiment. It was performed according to the Act on the Protection of Animals Used for Scientific or Educational Purposes in Poland adopted on 15 January, 2015, which complies with current EU regulations.

### 4.3. Blood Hormone Levels

Blood insulin and glucagon were assayed radioimmunologically using rat hormone RIA kits purchased from Merck Millipore (Darmstadt, Germany). Proinsulin and incretin hormones—glucose-dependent insulinotropic peptide (GIP) and glucagon-like peptide-1 (GLP-1) were determined by specific ELISA kits delivered by DRG Diagnostics (Marburg, Germany).

### 4.4. Western Blot Immunodetection

Total protein extracts of rat liver, the skeletal muscle and the visceral fat tissue were prepared using the T-PER Tissue Protein Extraction Reagent (Thermo Scientific, Waltham, MA, USA), with minor modifications of the recommended lysis procedure. The lysis buffer supplemented with protease and phosphatase inhibitor (Thermo Scientific, Rockford, IL, USA) was used to suspend tissue scraps (≈20 mg). They were homogenized on ice for 1 min, sonicated (50 W, 3 × 10 s), and centrifuged (14,000 rpm, 30 min). Protein concentration was determined by the Bradford method (Coomassie Plus Protein Assay Reagent-Thermo Scientific, Rockford, IL, USA) in received supernatants, and samples (10 μg protein/lane) were separated by sodium dodecyl sulfate-polyacrylamide gel electrophoresis. After separation, they were transferred (Mini-Protean Tetra Cell and Mini Trans-Blot systems, Bio-Rad, Hercules, CA, USA) to polyvinylidene difluoride membranes (Roche, Mannheim, Germany). Blots were washed, blocked with 5% skim milk, and incubated overnight at 4 °C with rabbit primary antibodies. Protein samples were tested with antibodies against insulin receptor β (1:4000), its phosphorylated fraction (p-IR β Tyr1150/1151 1:1000) (both purchased at Cell Signaling Technology Inc., Danvers, MA, USA), glucose transporter GLUT4 (1:3000, Thermo Fisher Scientific, Rockford, IL, USA) and Tug protein (1:15,000, Abcam, Cambridge, UK). Membranes were transferred to room temperature and washed, then incubated for 1 h with the peroxidase-conjugated anti-rabbit antibody (Jackson ImmunoResearch Laboratories, West Grove, PA, USA; 1:20,000). SuperSignal^®^ West Pico PLUS Chemiluminescent Substrate (Thermo Scientific Pierce, Carlsbad, CA, USA) was used to reveal immunoreactive bands. The membranes were then stripped (Restore^TM^ PLUS Western Blot Stripping Buffer, Thermo Scientific, Rockford, IL, USA) and reprobed with the anti-GAPDH or anti-β-actin antibody (1:30,000 and 1:10,000, respectively; Cell Signaling Technology Inc., Danvers, MA, USA). The Azure c300 Imaging System was used to detect signals and then they were quantified by densitometry using the AzureSpot v. 13.1 software (Azure Biosystems, Dublin, CA, USA). Tissues from each animal in each group were analyzed separately (*n* = 10).

### 4.5. Insulin Receptor Kinetics

Liver and skeletal muscle samples were prepared according to the method of Havrankova et al. [43]. Tissues pooled from 2–3 rats were homogenized in the ice cold buffer containing 1 mM NaHCO_3_ and centrifuged to obtain plasma membranes. The membrane protein content was determined by the BCA method with Pierce^TM^ BCA Protein Assay Kit (Thermo Scientific, Rockford, IL, USA). The final used concentration of protein was 0.5 mg/mL. The unlabeled insulin (Mixtard 50 Penfill, Novo Nordisk, Denmark) and ^125^I-labelled insulin (porcine) receptor grade (Perkin Elmer, specific activity 13.7 MBq/μg) were used as ligands. The incubations of ligands with plasma membranes were performed using a constant amount (0.03 nM) of labeled hormone and varying (from 0 to 715 nM) amounts of unlabeled one in a final volume of 0.5 mL 40 mM Tris-HCl buffer (pH 7.4) containing 0.1% BSA. 10.7 μM concentration of unlabeled insulin was used for nonspecific binding. The incubations were carried out for 18 h at 4 °C. The nonspecific binding of ^125^I-insulin was determined at 10 μM concentration of the unlabeled hormone. Bound and free fractions of insulin were separated by centrifugation at 20,000 *g* for 8 min. The radioactivity of pellets was measured using γ-counter (Wallac Wizard 1470 Gamma Counter). Two classes of insulin receptors were taken into account, i.e., high-affinity insulin receptors (HAIRs) and low-affinity insulin receptors (LAIRs). The binding capacities of two classes of receptors were determined by Scatchard’s method [44] using the program prepared by authors. The program was written in Python. The method described by Feldman [45] was implemented. The libraries built into the Python language, NumPy, and SciPy for mathematical and scientific calculations, were used.

The dissociation constant for HAIRs (Kd_1_) and for LAIRs (Kd_2_), as well as the maximum binding capacity of HAIRs (R_1_) and of LAIRs (R_2_) were determined in the liver and in the skeletal muscle. The results were calculated per 1 mg of membrane protein. The bicinchoninic acid (BCA) assay test was used to determine protein content in tissue homogenates (Thermo Scientific, Rockford, IL, USA).

### 4.6. Statistical Analysis

The obtained results (except for insulin binding) were expressed as means ± standard error of the mean (SEM) of 10 rats and were evaluated statistically by one-way ANOVA and Tukey’s multiple comparison test. In the case of studies concerning insulin binding, tissues from 2–3 rats were pooled, and results were expressed as the means ± SEM from 4 separate determinations and were evaluated statistically by the non-parametric Mann–Whitney test. The test was used to compare differences between GKC and GKR, and also SDC and GKC. For all results, the GraphPad Prism for Windows software was applied (license no. GRA/3802/2015, La Jolla, CA, USA). Differences were considered statistically significant at *p* < 0.05.

## 5. Conclusions

In conclusion, GK rats have insulin resistance with concomitant hyperinsulinemia. This is associated with a substantial dysregulation of insulin signaling and with disturbances covering many diabetes-related parameters. Resveratrol was shown to alleviate some disturbances in GK rats. The most relevant finding is the normalization of the insulin receptor expression level in the skeletal muscle and also proteins related to intracellular glucose transport (GLUT4 and TUG) in adipose tissue. Moreover, parameters concerning insulin binding were also affected by resveratrol. These changes may be supposed to have positive effects on metabolism.

## Figures and Tables

**Table 1:** Effects of resveratrol on blood hormone levels in SD and GK rats.

Parameter	SDC	SDR	GKC	GKR
Insulin (ng/mL)	2.01 ± 0.27	2.51 ± 0.39	5.48 ± 0.53 *,#	6.2 ± 0.63 *,#
Proinsulin (pmol/mL)	42.37 ± 1.6	43.20 ± 0.9	22.51 ± 0.8 *,#	23.35 ± 6.7 *,#
Glucagon (pg/mL)	115 ± 12.3	103 ± 8.7	122 ± 16.4	113 ± 13.4
GIP (ng/mL)	14.26 ± 0.62	13.87 ± 0.45	8.35 ± 0.49 *,#	7.99 ± 0.35 *,#
GLP-1 (ng/mL)	0.58 ± 0.02	0.53 ± 0.03	0.74 ± 0.06 #	0.71 ± 0.05 #

GIP—glucose-dependent insulinotropic peptide, GLP-1—glucagon-like peptide-1, SDC—Sprague-Dawley control rats, SDR—Sprague-Dawley rats treated with resveratrol, GKC—Goto-Kakizaki control rats, GKR—Goto-Kakizaki rats treated with resveratrol. Results represent the means ± SEM from 10 rats. Differences statistically significant (*p* < 0.05) *—compared with SDC group, # compared with SDR group.

**Table 2:** Effects of resveratrol on parameters related to insulin binding in GK and SD rats.

Parameter	SDC	SDR	GKC	GKR
Kd_1_ Liver (pM)	1.028 ± 0.211 *	2.206 ± 0.560	2.570 ± 0.373	4.658 ± 0.561 *
Kd_2_ Liver (pM)	513.858 ± 44.447 *	518.362 ± 79.162	295.119± 75.735	798.021 ± 175.886 *
R_1_ Liver (pM)	48.006 ± 11.211	23.154 ± 7.192	30.897 ± 2.499	9.240 ± 1.378 *
R_2_ Liver (pM)	0.190 ± 0.017	0.287 ± 0.054	0.335 ± 0.109	0.114 ± 0.032 *
Kd_1_ Skeletal muscle (fM)	4.247 ± 1.431	1.238 ± 0.008	2.791 ± 0.567	0.647 ± 0.074 *
Kd_2_ Skeletal muscle (fM)	3636.828 ± 61.378	2592.685 ± 64.992	5487.313 ± 114.611	406.922 ± 44.274 *
R_1_ Skeletal muscle (fM)	9038.727 ±3511.087	7789.672 ± 89.998	3963.148 ± 425.352	14137.999 ± 427.379 *
R_2_ Skeletal muscle (fM)	8.175 ± 0.075 *	8.807 ± 0.045	3.953 ± 1.046	17.973 ± 2.517 *

Kd_1_—dissociation constant for HAIRs, Kd_2_—dissociation constant for LAIRs, R_1_—maximum binding capacity of HAIRs (Bmax for HAIRs), R_2_—maximum binding capacity of LAIRs (Bmax for LAIRs), SDC—Sprague-Dawley control rats, SDR—Sprague-Dawley rats treated with resveratrol, GKC—Goto-Kakizaki control rats, GKR—Goto-Kakizaki rats treated with resveratrol. Tissues from 2–3 rats were pooled, and results represent the means ± SEM from 4 separate determinations. *—Differences statistically significant (*p* < 0.05) compared with GKC group.

## Data Availability

Data is contained within the article.

## Supplementary Materials

The following are available online at https://www.mdpi.com/1422-0067/22/5/2469/s1, Figure. All images of western blots for Figure 2C, Figure 3B, Figure 4E and Figure 4F.

## Abbreviations

ACC	Acetyl-CoA carboxylase
Akt	Protein kinase B
FIGR	Fasting insulin/glucose ratio
GIP	Glucose-dependent insulinotropic peptide
GKC	Goto-Kakizaki control rats
GKR	Goto-Kakizaki rats treated with resveratrol
GLP-1	Glucagon-like peptide-1
GLUT4	Glucose transporter 4
HAIRs	High-affinity insulin receptors
HOMA-IR	Homeostatic model assessment—insulin resistance
IDE	Insulin-degrading enzyme
IRS-1	Insulin receptor substrate-1
Kd_1_	Dissociation constant for HAIRs
Kd_2_	Dissociation constant for LAIRs
LAIR	Low-affinity insulin receptors
QUICKI	Quantitative insulin sensitivity check index
R_1_	Maximum binding capacity of HAIRs
R_2_	Maximum binding capacity of LAIRs
SDC	Sprague-Dawley control rats
SDR	Sprague-Dawley rats treated with resveratrol
TUG	Tether containing a UBX domain for GLUT4
