# Supplementary material for: Resveratrol Affects Insulin Signaling in Type 2 Diabetic Goto-Kakizaki Rats
Source: Int J Mol Sci. 2021 Feb 28;22(5):2469. doi: 10.3390/ijms22052469 (PMC7957525; doi:10.3390/ijms22052469)

Figure S1:

IR (muscle)

The red frames on photos below indicate the 3 representatives of each group of rats, which are presented on figure 2C in the article.

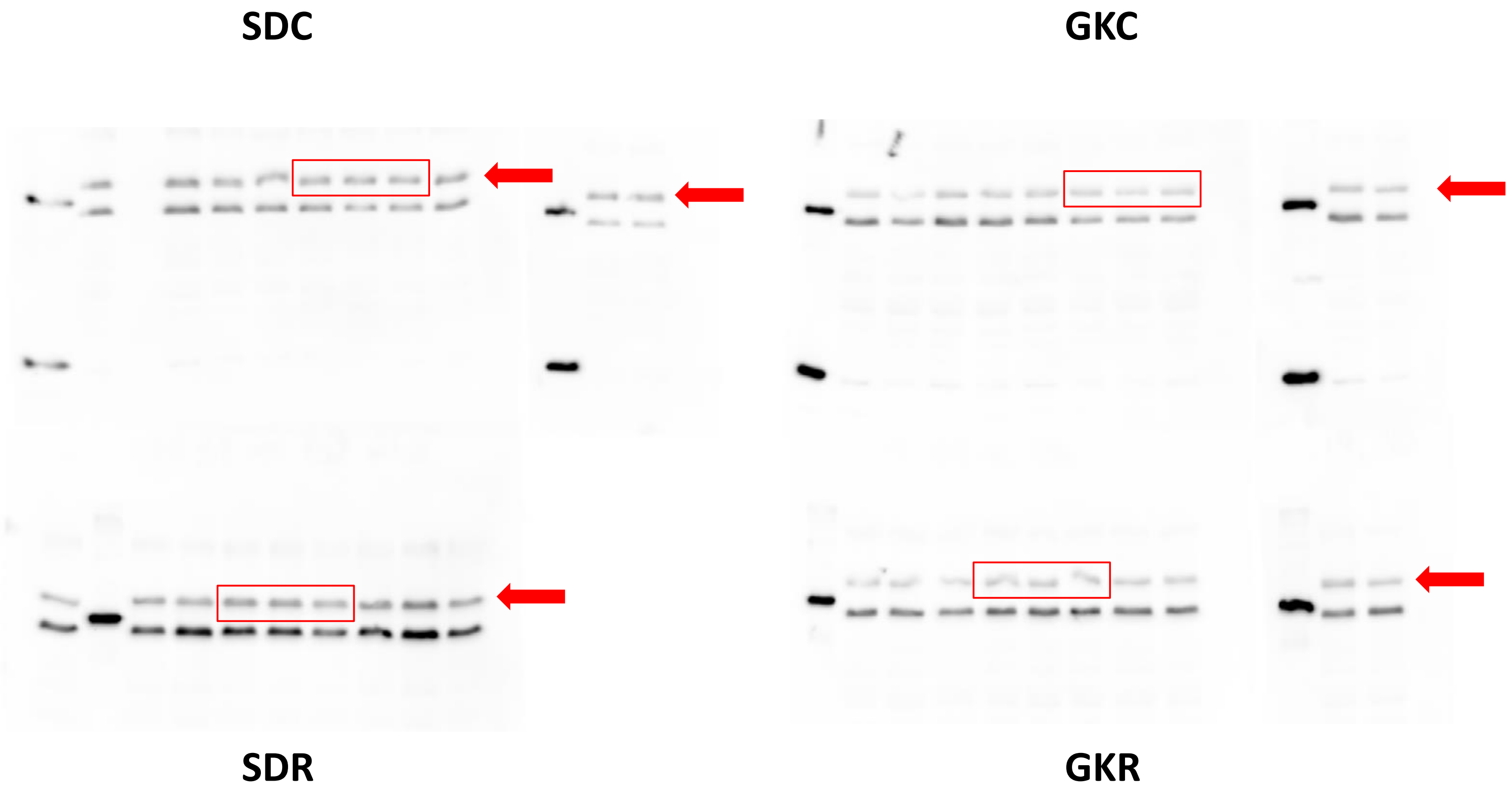

pIR (muscle)

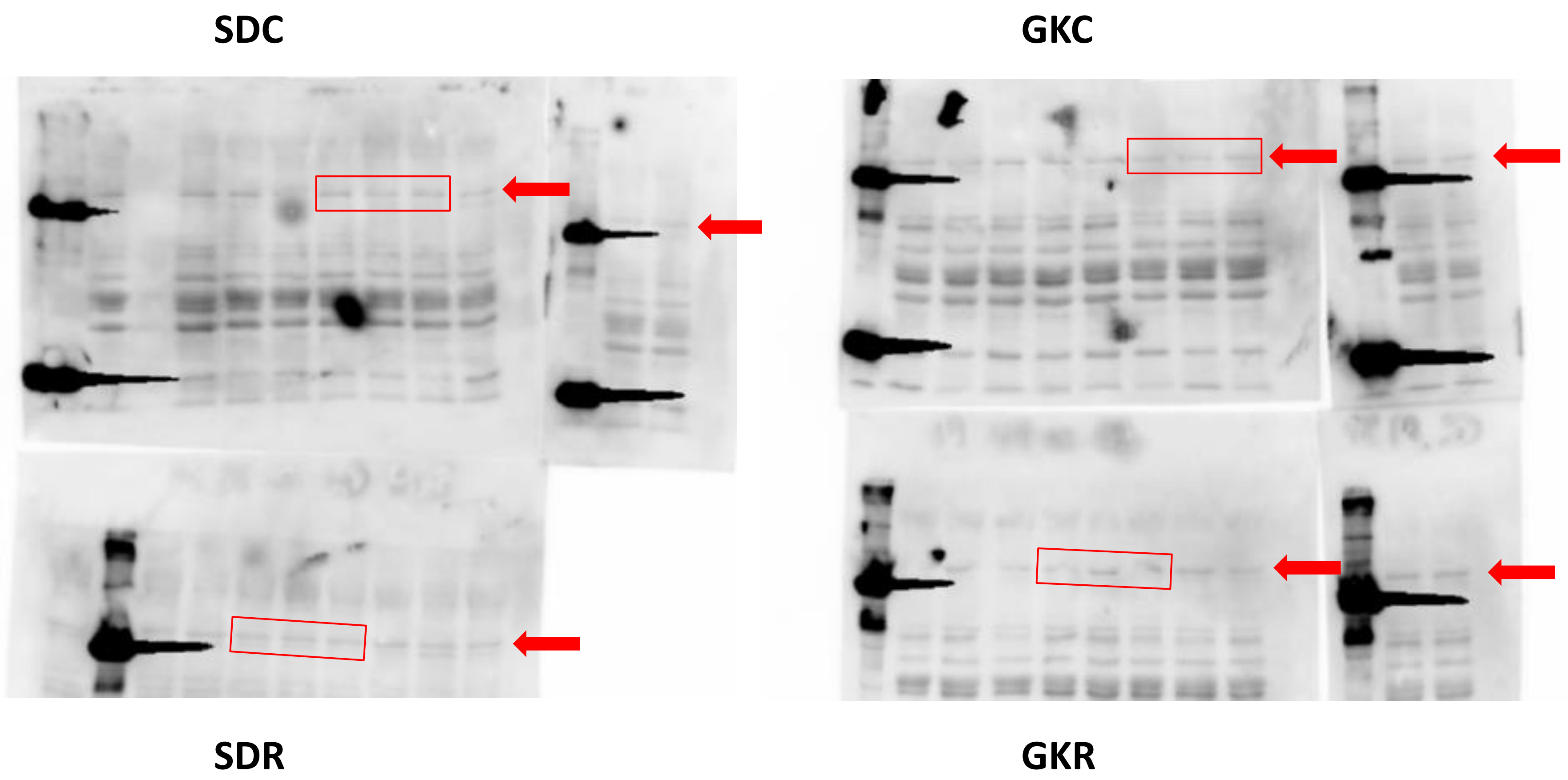

GAPDH (muscle)

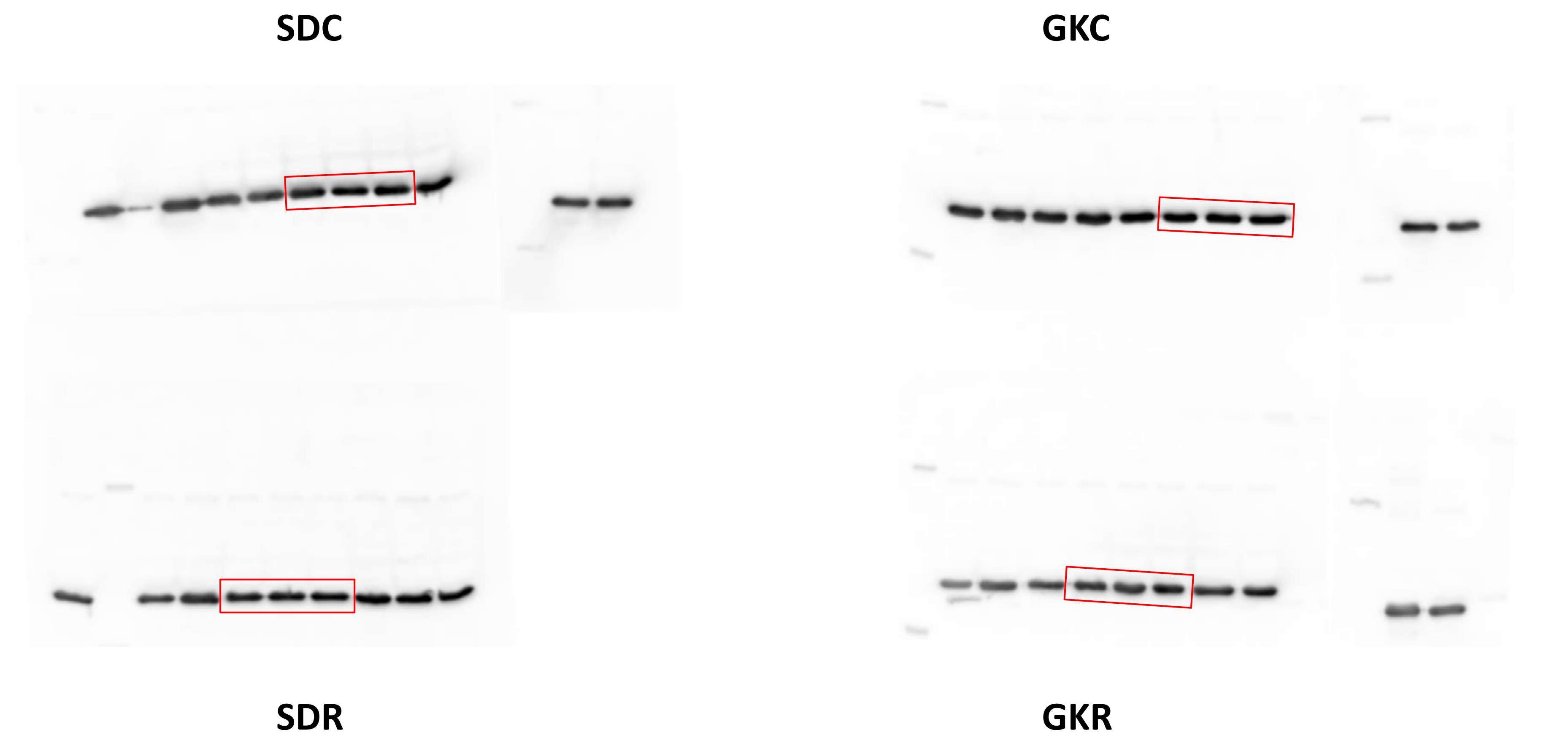

Figure S2:

IR (liver)

The red frames on photos below indicate the 3 representatives of each group of rats, which are presented on figure 3B in the article.

SDC **GKC**

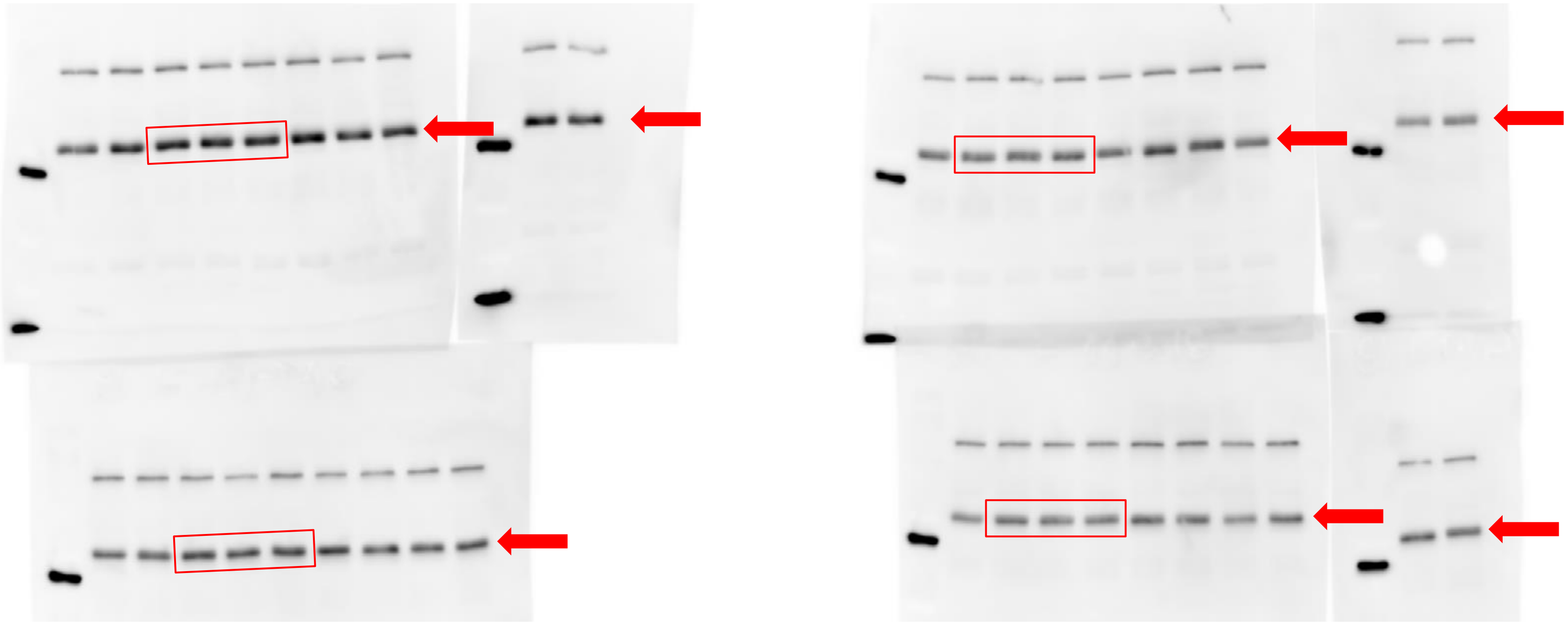

SDR **GKR**

β actin (liver)

SDC **GKC**

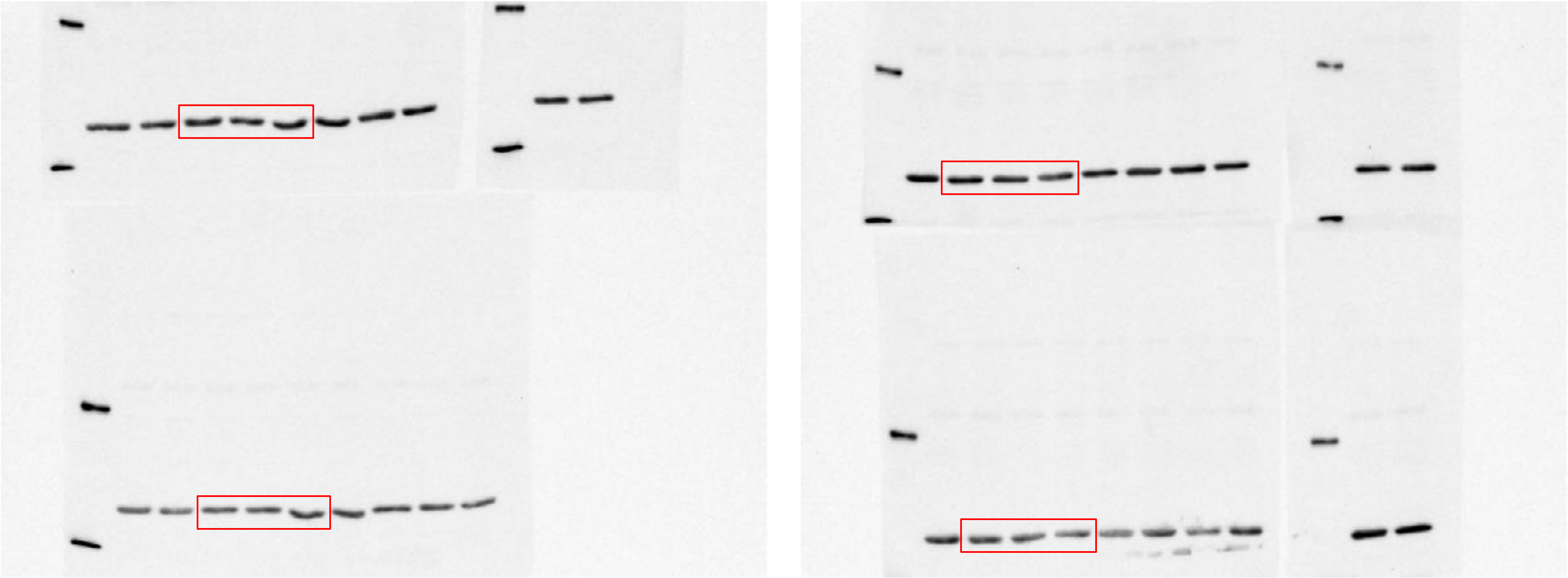

SDR **GKR**

Figure S3A: Tug (muscle)

The red frames on photos below indicate the 3 representatives of each group of rats, which are presented on figure 4E in the article.

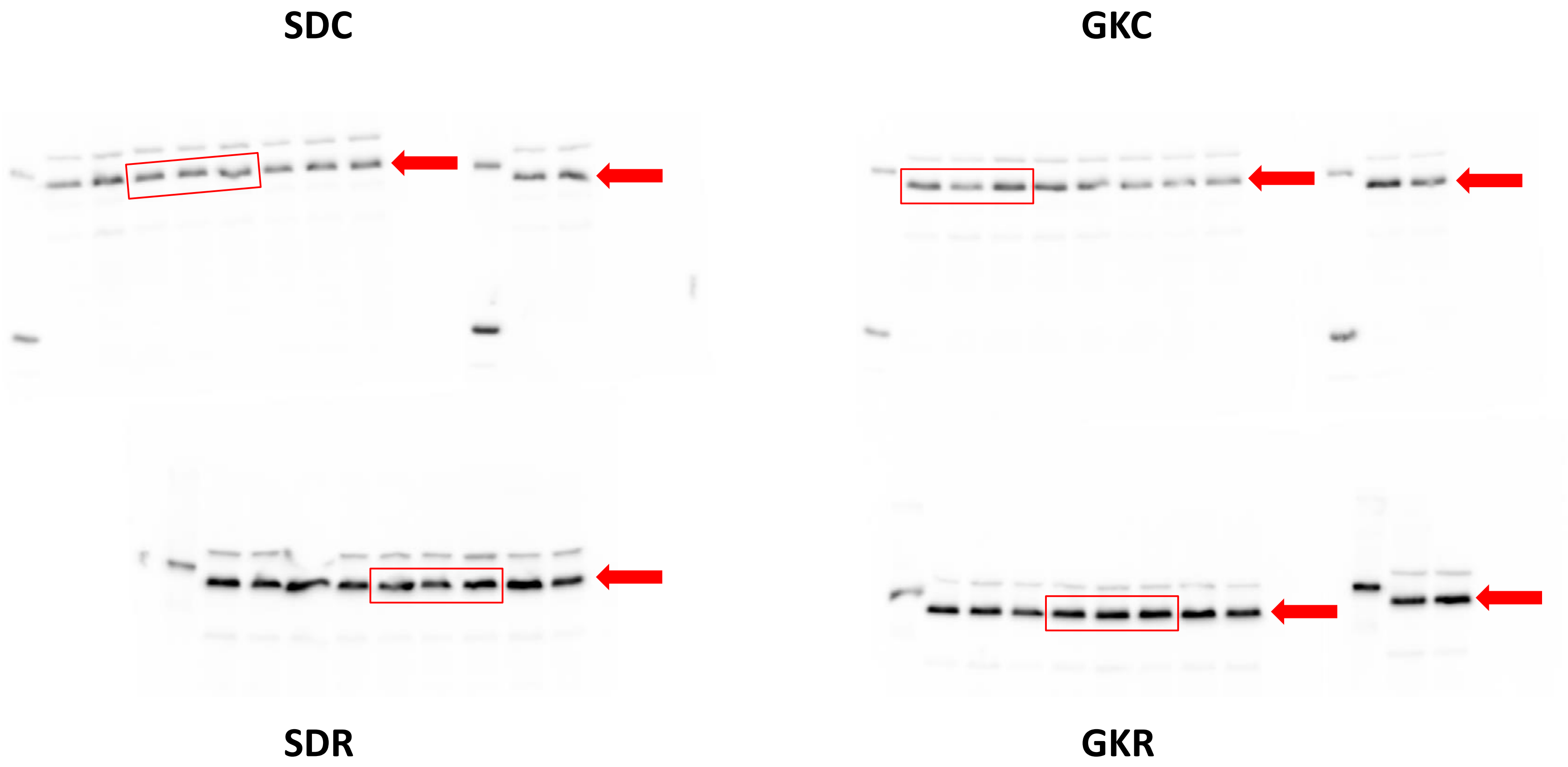

Glut4 (muscle)

SDC

GKC

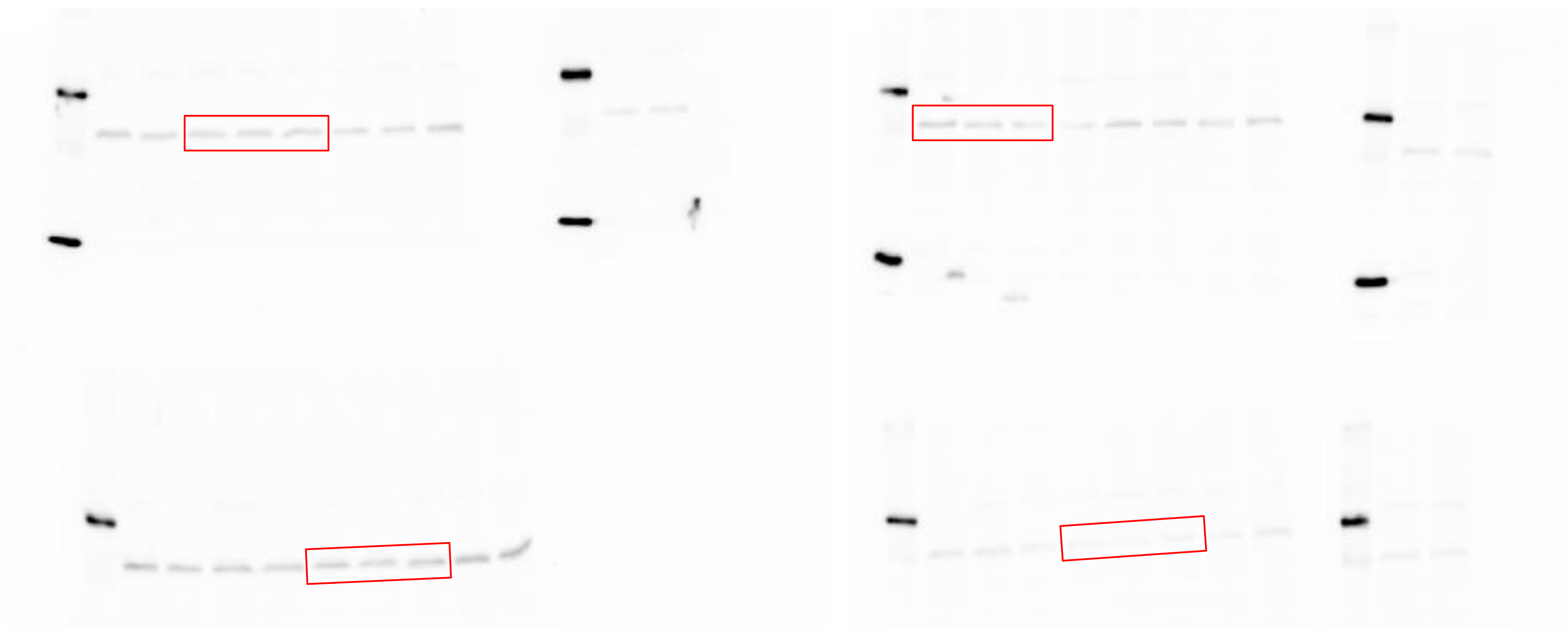

SDR

GKR

GAPDH (muscle)

SDC

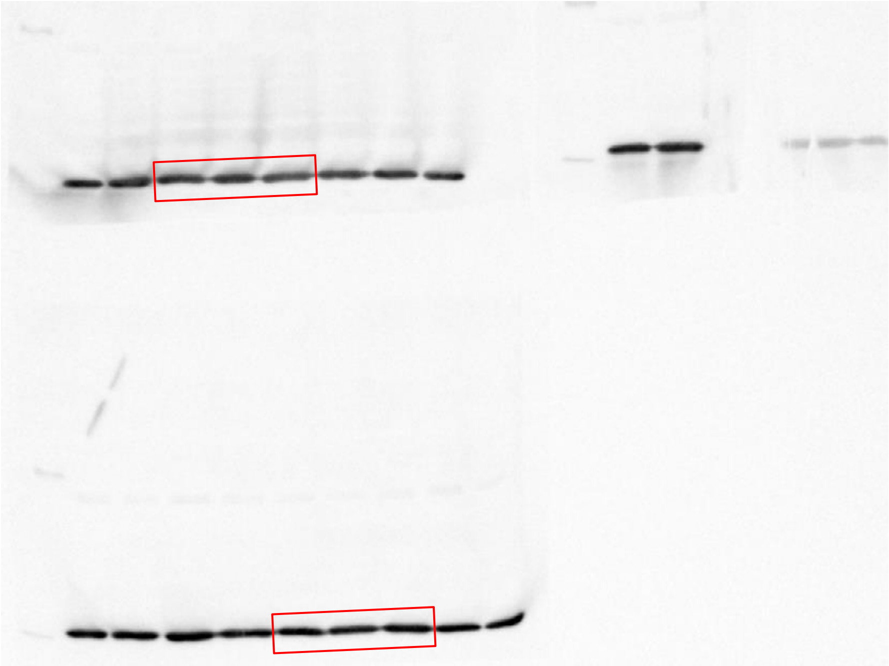

SDR

GKC

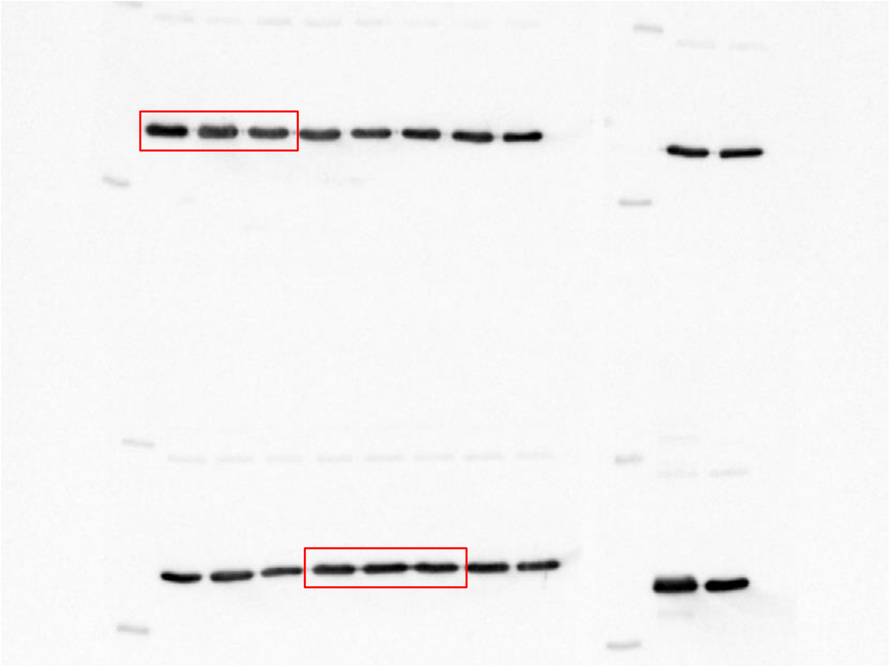

Figure S3B:

Tug (fat)

The red frames on photos below indicate the 3 representatives of each group of rats, which are presented on figure 4F in the article.

SDC **GKC**

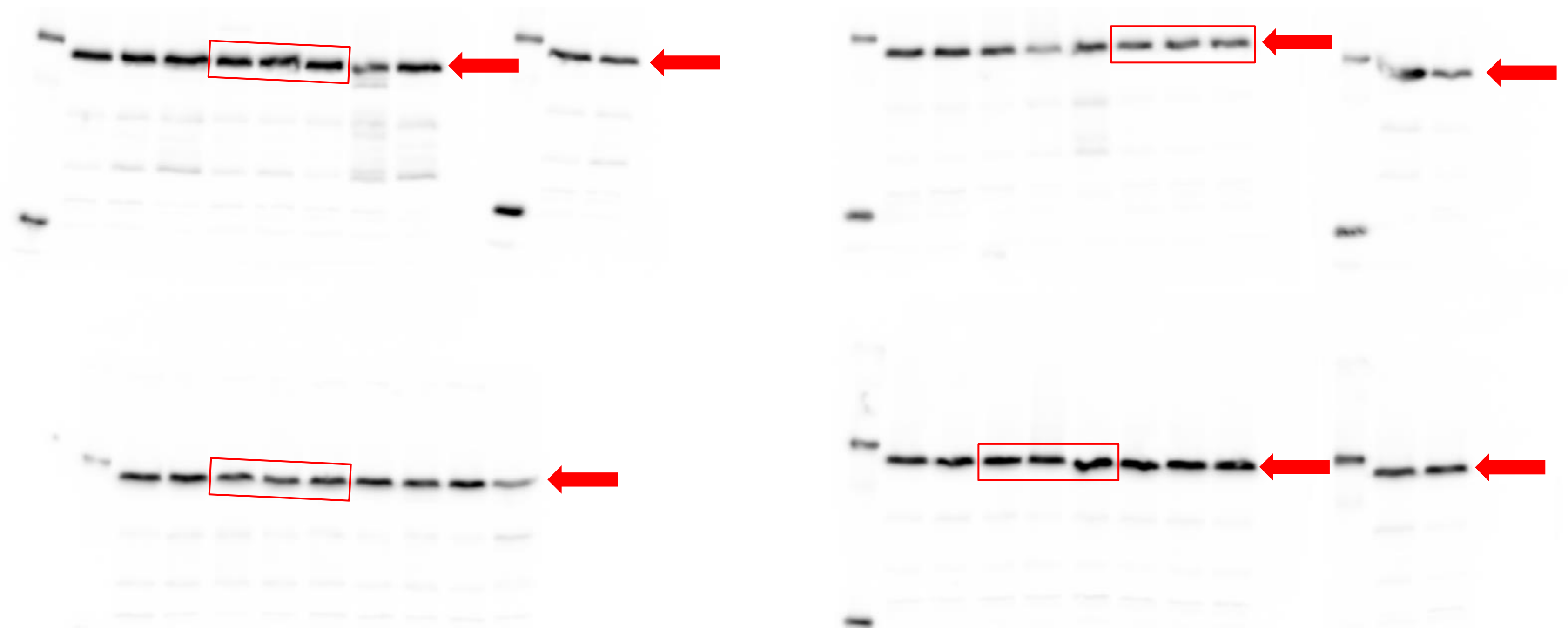

SDR **GKR**

Glut4 (fat)

SDC **GKC**

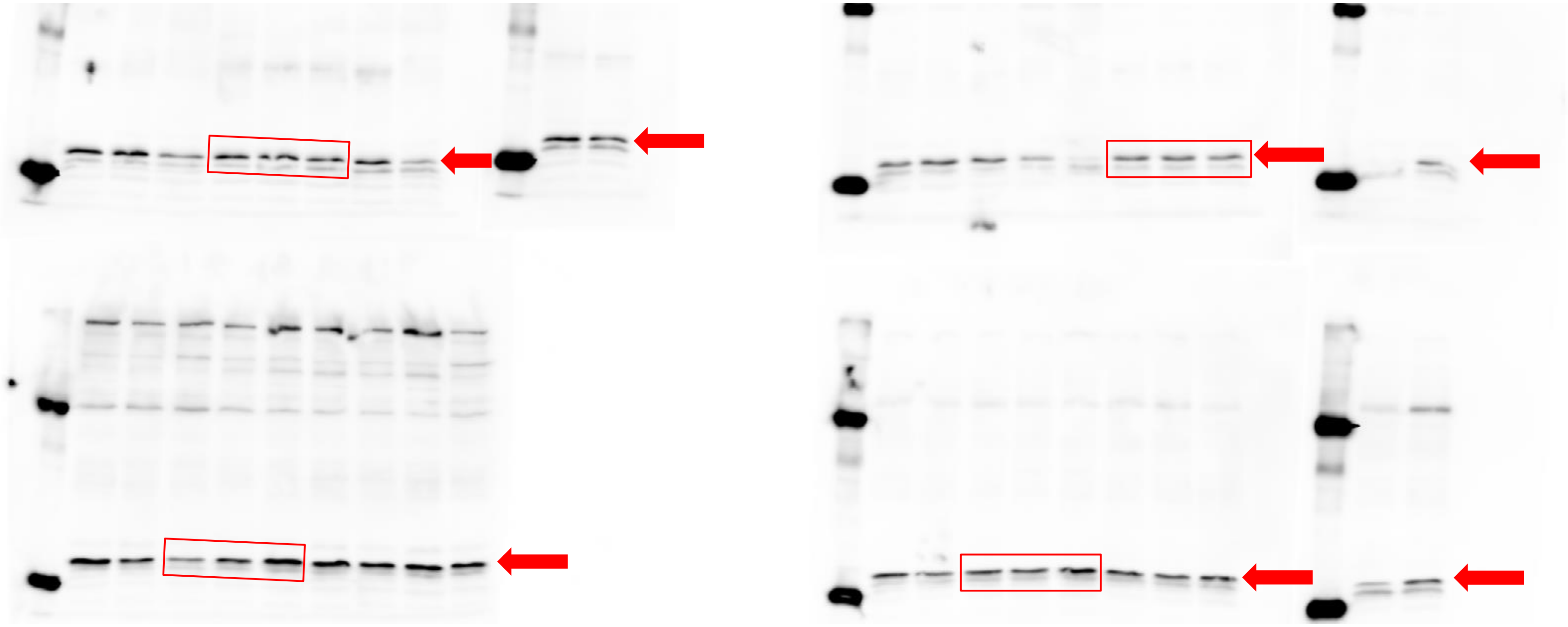

SDR **GKR**

β actin (fat)

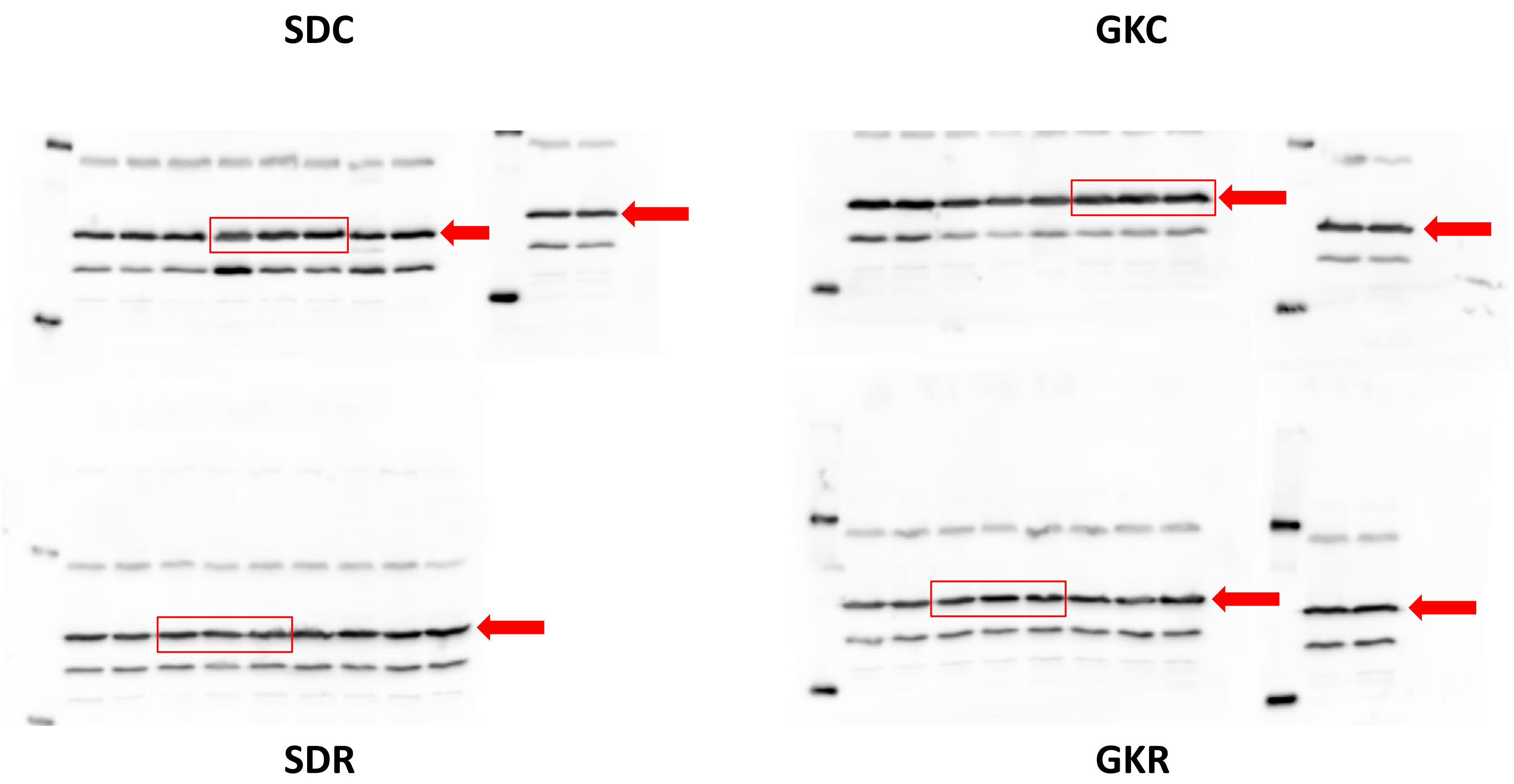

Supplement: Supplementary file 1 [file ijms-22-02469-s001.pdf]
